# Delayed Replantation after Endodontic and Fluoride Treatment: A 5-Year Follow-up

**DOI:** 10.5005/jp-journals-10005-1114

**Published:** 2011-04-15

**Authors:** Paul Chalakkal, Abi Mathew Thomas, Francis Akkara, Kristlee Sabrin Fernandes

**Affiliations:** 1 Lecturer, Department of Pedodontics and Preventive Dentistry, Goa Dental College and Hospital, Goa, India; 2 Professor, Department of Pedodontics and Preventive Dentistry, Christian Dental College and Hospital, Punjab, India; 3 Assistant Professor, Department of Oral and Maxillofacial Surgery, Goa Dental College and Hospital, Bambolim, Goa, India; 4 Lecturer, Department of Conservative Dentistry and Endodontics, Goa Dental College and Hospital, Bambolim, Goa, India

**Keywords:** Delayed replantation, Avulsion, Fluoride.

## Abstract

**Aim:**

To evaluate if endodontic and fluoride treatment of the root before delayed replantation would render the tooth a favorable prognosis.

**Methods:**

A 10-year-old boy reported 10 hours after he had avulsed his maxillary right permanent central incisor. The pulp and PDL cells were considered to be nonviable. Endodontic treatment for the tooth was performed extraorally and obturated with gutta-percha. Prior to replantation, the root was treated with 1.23% APSF for 15 minutes.

**Result:**

An intraoral radiograph taken 6 months after replantation revealed narrowing of the PDL space around the replanted tooth due to apposition from the surrounding alveolar bone. A radiograph taken 5 years after replantation revealed no evidence of external root resorption. There was no abnormal mobility either.

**Conclusion:**

Extraoral endodontic treatment and root treatment with 1.23% APSF prior to delayed replantation might prevent the occurrence of external root resorption.

## INTRODUCTION

It is often impossible for the patient to report immediately after avulsion has occurred owing to lack of knowledge, physical health impairment, mental confusion and travel time. In most cases, the use of inappropriate transport media worsen the chances of pulp and periodontal survival.

Andreasen claimed that replacement resorption occurred if the extraoral dry time was more than 60 minutes and no cell remained vital beyond 120 minutes of dry time on the root.^1^ Lekic and Mcculloh demonstrated *in vitro* that beyond 15 minutes, progenitor periodontal ligament cells exhibited limited proliferative ability, which became impossible after 30 minutes.^2^ Recently, the time period for immediate replantation has changed from 30 minutes to less than 5 minutes of extraoral time.^3^

The aim of this case report was to evaluate if extraoral endodontic and fluoride treatment of the root before delayed replantation would render the tooth a favorable prognosis.

## METHODS

**Fig. 1:**
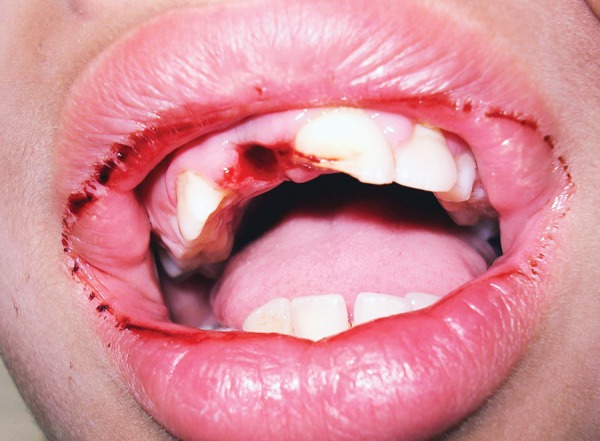
Intraoral view of the avulsion site

**Fig. 2:**
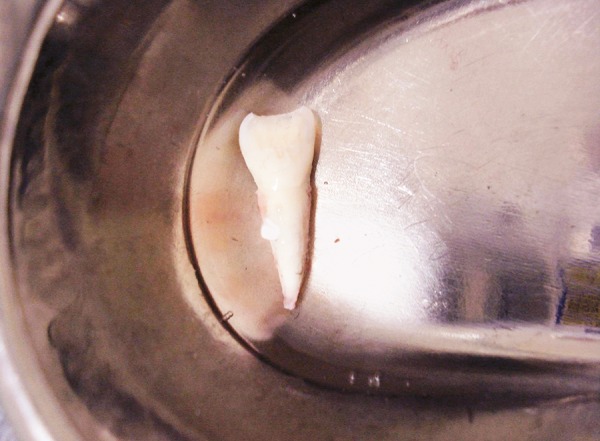
Tooth placed in 1% sodium hypochlorite

**Fig. 3:**
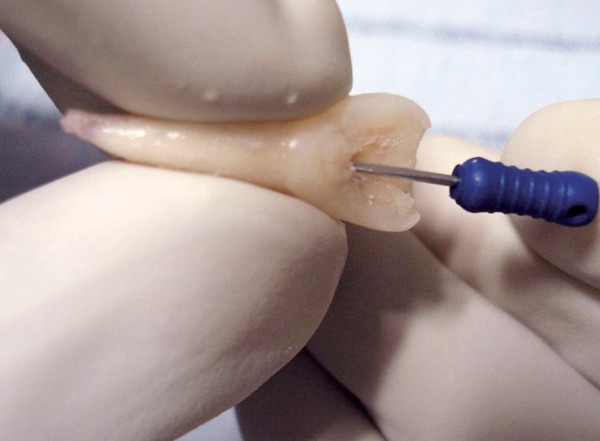
Extraoral endodontic treatment in the avulsed tooth

**Fig. 4:**
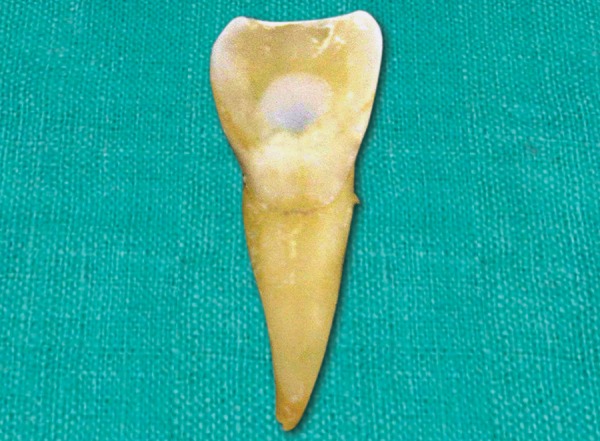
Access cavity sealed with composite

**Fig. 5:**
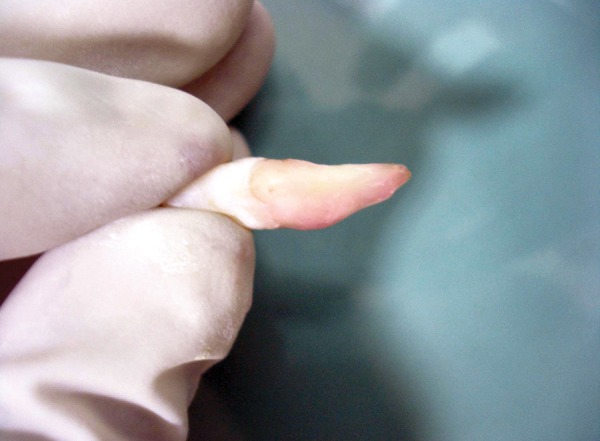
Root treatment with 1.23% APSF

**Fig. 6:**
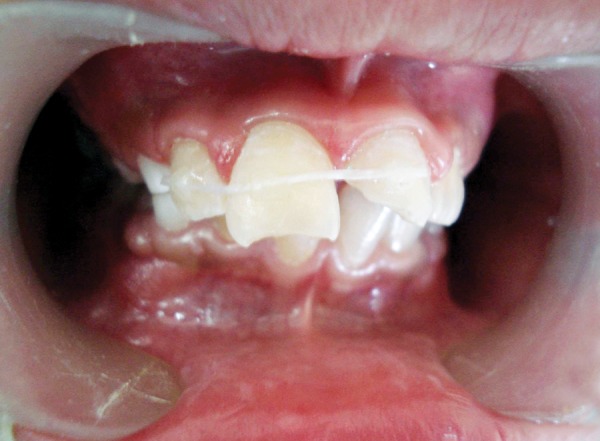
Floss and composite splint

**Fig. 7:**
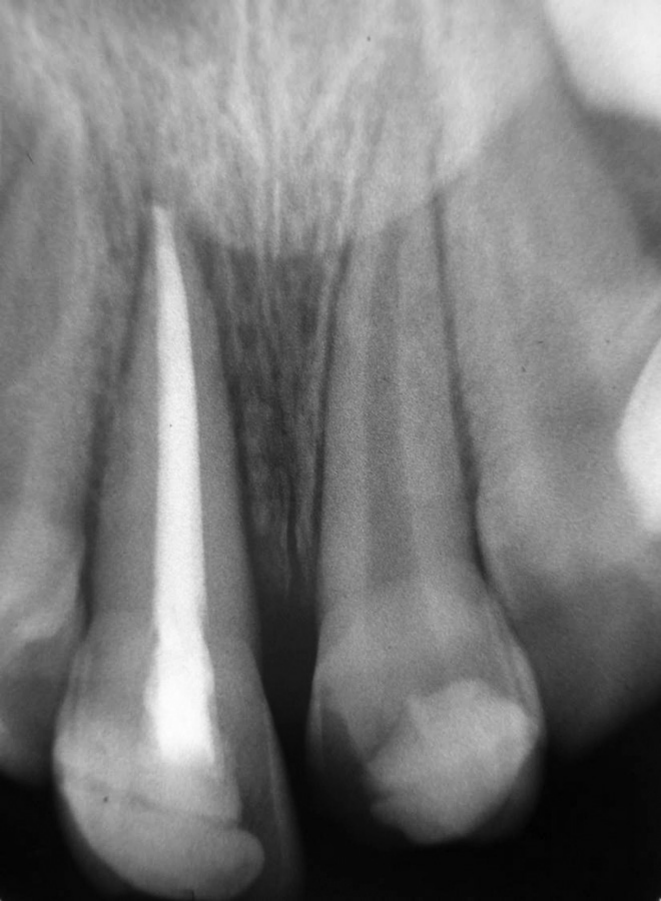
Intraoral radiograph soon after replantation

A 10-year-old boy reported to the dept of pedodontics and preventive dentistry 10 hours after he had avulsed his maxillary right permanent central incisor after a fall in school. He had preserved the tooth in his buccal vestibule all the while. On examination, there were no extraoral signs or symptoms and the boy was conscious and mobile. There were no apparent intraoral lacerations or alveolar fractures (Fig. 1). Due to a time lag of 10 hours, the pulp and PDL cells were considered to be nonviable. The tooth was placed in 1% sodium hypochlorite solution to dissolve PDL remnants (Fig. 2), after which, the root surface was scraped with cotton soaked in the same solution. Endodon-tic treatment for the tooth was performed extraorally (Fig. 3). Obturation was done with gutta percha and the access cavity was sealed with composite (Fig. 4). Prior to replantation, the root was treated with 1.23% APSF for 15 minutes (Fig. 5). The socket was flushed with saline to clear any blood clots. The tooth was replanted under local anesthesia and splinted with floss and composite (Fig. 6). An intraoral periapical radiograph was taken to ensure the replanted position (Fig. 7). A loading dose of 1000 mg amoxicillin was administered immediately, to be followed with 500 mg amoxycillin thrice daily for 7 days. He was advised a soft diet and tooth brushing after every meal. Chlorhexidine mouthwash (0.2%) was prescribed to be used twice daily for a week. A tetanus prophylactic was also administered.

## RESULTS

**Fig. 8:**
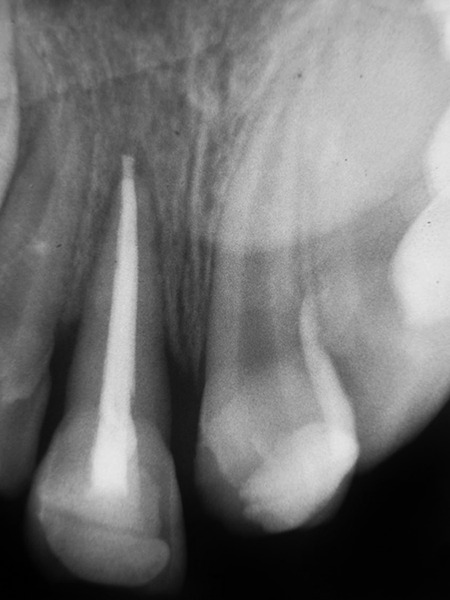
Intraoral radiograph―6 months after replantation

**Fig. 9:**
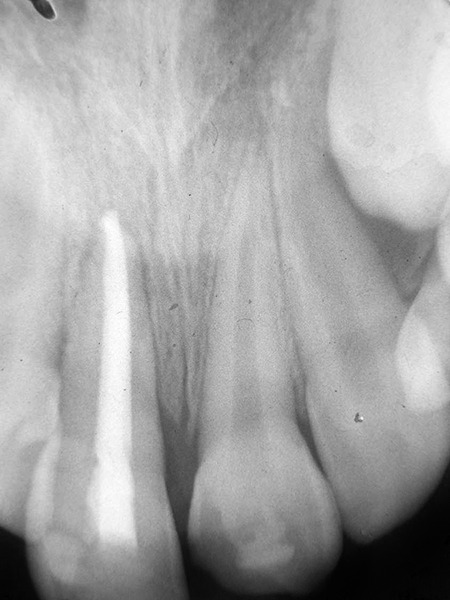
Intraoral radiograph―40 months after replantation

There was absence of pain or swelling after replantation with no extraoral signs or symptoms. An intraoral radiograph taken 6 months after replantation revealed narrowing of the PDL space around the replanted tooth due to apposition from the surrounding alveolar bone (Fig. 8). A radiograph taken 5 years after replantation revealed no particular difference from the previous radiograph (Fig. 9). The tooth expressed no abnormal mobility. There was no evidence of root resorp-tion, radiographically.

## DISCUSSION

Bacteria in the root canal (or dentinal tubules) and cementum (or PDL cells) trigger inflammatory root resorption.^4^ The factors responsible for prevention of replacement resorp-tion are cementoblasts, precementum and epithelial rests of Malassez.^5^ The replacement of cementoblasts by osteoblasts, which have a receptor for the parathyroid hormone, together with the bone-cementum (or dentin) interface, allows the tooth to participate in the bone remodeling process resulting in its gradual replacement with bone.^6^

Root treatment without the removal of PDL fibres results in ankylosis or replacement resorption.^7^ Sodium hypochlorite preserves the integrity of cementum and has been used for the removal of PDL fibres from avulsed teeth before delayed replantation.^8^

Fluoride, apart from its antimicrobial property, acts on cementum and dentin converting hydroxyapatite into fluoroapatite which is more resistant to resorption, or even inhibits clastic cell formation.^7^

As per guidelines for avulsed teeth with completely formed roots that are replanted after an extraoral time beyond 60 minutes, the International Dental Traumatology Association has suggested that these teeth, after much removal of PDL remnants, should be immersed in 2.4% APSF (pH 5.5) for 5 minutes.^9^

Endodontic treatment is required for replanted teeth because the necrotic pulp and its toxins affect the PDL through dentinal tubules causing resorption.^10^

In this case report, although there has not been any evidence of ankylosis or replacement resorption, it does not necessarily guarantee a good prognosis for the tooth. Even if replacement resorption begins to occur, tooth loss would occur gradually without loss of alveolar height which is important if a prosthesis is to be considered in the future. In that case, gutta percha would have to be withdrawn in order to encourage the process and to promote bone filling.

## CONCLUSION

After 5-year follow-up evaluation and delayed replantation, it can be concluded that:

- Root treatment (after denuding the root of PDL fibres) with 1.23% APSF prior to replantation might prevent the occurrence of external root resorption
- Performing endodontic treatment extraorally might prevent the occurrence of external root resorption.

## References

[1] Andreasen JO (1981). Effect of extra-alveolar period and storage media upon periodontal and pulpal healing after replantation of mature permanent incisors in monkeys.. Int J Oral Surg.

[2] Lekic P, McCulloch CA (1996). Periodontal ligament cell populations: The central role of fibroblasts in creating a unique tissue.. Anat Rec.

[3] Kenny DJ, Barrett EJ (2001). Recent developments in dental traumatology.. Pediatr Dent.

[4] Finucane D, Kinirons MJ (2003). External infammatory and replacement resorption of luxated, and avulsed replanted permanent incisors: A review and case presentation.. Dent Traumatol.

[5] Wallace JA, Vergona K (1990). Epithelial rests function in replantation: Is splinting necessary in replantation?. Oral Surg Oral Med Oral Pathol.

[6] Consolaro A. (2002). Dental resorptions in the clinic specialties..

[7] Shulman LB, Gedalia I, Feingold RM (1973). Fluoride concentration in root surfaces and alveolar bone of fluoride immersed incisors three weeks after replantation.. J Dent Res.

[8] Panzarini SR, Perri de Carvalho AC, Poi WR, Sonoda CK (2005). Use of vitamin C in delayed tooth replantation.. Braz Dent J.

[9] Flores MT, Andreasen JO, Bakland LK, Feiglin B, Gutmann JL, Oikarinen K, Pitt Ford TR, Sigurdsson A, Trope M, Vann WF Jr (2001). Guidelines for the evaluation and management of traumatic dental injuries.. Dent Traumatol.

[10] Andreasen JO, Borum MK, Jacobsen HL, Andreasen FM (1995). Replantation of 400 avulsed permanent incisors 4. Factors related to periodontal ligament healing.. Endod Dent Traumatol.

